# *RAB39B* gene mutations are not linked to familial Parkinson’s disease in China

**DOI:** 10.1038/srep34502

**Published:** 2016-10-03

**Authors:** Ji-feng Kang, Yang Luo, Bei-sha Tang, Chang-min Wan, Yang Yang, Kai Li, Zhen-hua Liu, Qi-ying Sun, Qian Xu, Xin-xiang Yan, Ji-feng Guo

**Affiliations:** 1 Department of Neurology, Xiangya Hospital, Central South University, Changsha, 410008 Hunan, People’s Republic of China; 2 State Key Laboratory of Medical Genetics, Changsha, 410008 Hunan, People’s Republic of China; 3 Key Laboratory of Hunan Province in Neurodegenerative Disorders, Central South University, Changsha, 410008 Hunan, People’s Republic of China; 4 Neurodegenerative Disorders Research Center, Central South University, Changsha, 410008 Hunan, People’s Republic of China

## Abstract

Recently, *RAB39B* mutations were reported to be a causative factor in patients with Parkinson’s disease (PD). To validate the role of *RAB39B* in familial PD, a total of 195 subjects consisting of 108 PD families with autosomal-dominant (AD) inheritance and 87 PD families with autosomal-recessive (AR) inheritance in the Chinese Han population from mainland China were included in this study. We did not identify any variants in the coding region or the exon-intron boundaries of the gene by Sanger sequencing method in the DNA samples of 180 patients (100 with AD and 80 with AR). Furthermore, we did not find any variants in the *RAB39B* gene when Whole-exome sequencing (WES) was applied to DNA samples from 15 patients (8 with AD and 7 with AR) for further genetic analysis. Additionally, when quantitative real-time PCR was used to exclude large rearrangement variants in these patients, we found no dosage mutations in *RAB39B* gene. Our results suggest that *RAB39B* mutation is very rare in familial PD and may not be a major cause of familial PD in the Chinese Han Population.

Parkinson’s disease (PD) is a common late onset neurodegenerative disorder, clinically characterized by a series of motor symptoms and non-motor symptoms. The classical motor symptoms such as bradykinesia, rigidity, rest tremor, and postural instability have been widely recognized as prominent components of PD^1^. PD is a complex disease with a combination of environmental exposures as well as the effect of multiple gene mutations. Previous studies reported that about 10% of the patients have a positive family history of PD and that a series of genes, such as *SNCA*, *LRRK2*, *VPS35*, *Parkin*, *PINK1*, and *DJ-1*, were associated with the disease^2^.

A recent publication by Wilson *et al.* reported that mutations of the *RAB39B* gene were responsible for the cause of X-linked intellectual disability and early-onset PD in two unrelated families that come from Australian and the USA (Wisconsin)^3^. Their study identified a ~45 kb deletion resulting in complete deletion of the *RAB39B* gene in family A. A missense mutation, c.503C > A(p.Thr168Lys) in the *RAB39B* gene, was found in family B, which had been described 30 years ago^4^. The Threonine residue was shown to be evolutionarily-conserved^3^. In another group, Mata *et al.* reported a missense mutation, c.574G > A (p.Gly192Arg), in a North American family of European origin; the affected family members were characterized with a classical PD phenotype. This mutation was predicted to be deleterious as defined by Combined Annotation Dependent Depletion (CADD)^5^. More recently, Lesage *et al.*, reported a new truncating mutation (p.W186stop) in the *RAB39B* gene in a PD patient of French origin^6^. Previous studies have suggested that the *RAB39B* gene is causative of familial PD in different ethnic groups. To validate the role of the *RAB39B* gene in susceptibility to familial PD in the Chinese Han population, we conducted this study in 195 families consisting of PD patients with AD and AR inheritance, in whom the common causative genetic mutations, in genes such as *SNCA*, *LRRK2*, *UCHL1*, *HtrA2*, *GIGYF2*, *EIGIF4*, *parkin*, *PINK1*, *DJ-1*, *ATP13A2*, and *PLA2G6*, had previously been excluded^7^,^8^,^9^.

## Methods

### Patients

All the patients were treated in the Xiangya Hospital Neurology department from January 1995 to May 2015 throughout the study. Written informed consent was obtained from each subject. The work received approval from the institutional ethics committee of Xiangya hospital. We conducted this study in 108 AD inherited PD patients (mean age at onset, 47.82 ± 11.6 years, 57% males) and 87 AR inherited PD patients (mean age at onset, 49.19 ± 15.05 years, 55.7% males) from the Chinese Han population. Patients who had a positive family history of PD (2 or more patients in 1 family) were diagnosed by at least 2 professional neurologists according to the United Kingdom PD Brain Bank Criteria^10^.

### Genetic analysis

Genomic DNA from patients was extracted from peripheral blood by using standard protocols^8^. Polymerase chain reaction (PCR) analysis of the *RAB39B* gene was carried out by using 2 pairs of primers. The 2 pairs of primers were designed to cover the whole coding region and exon-intron boundaries of the *RAB39B* gene (the details of primer sequences are listed in Table 1), and 180 probands were genotyped by Sanger sequencing. The analysis of an additional 15 individuals from unrelated families (8 with AD and 7 with AR inheritance) was performed using Whole-exome sequencing (Beijing Genomics institution, BGI, China). The mean of 90% of exome target region reads had coverage of, at least 30× for further genetic analysis. The SureSelect Human All ExonV5 Kit (Agilent,USA) was used for exome targeted regions capture. Subsequently, the captured regions were sequenced using Hiseq 2000 platform (Illumina, CA) and aligned to the reference human genome database hg19. Burrows-Wheeler Aligner (BWA), Picard tools and Genome Analysis ToolKit (GATK) tools were used for sequence data alignment, duplicate reads assess and variation detection, respectively^11^. Then, the detected variations were catalogued by Annovar, and common variations were filtered with dbSNP, 1000 Genomes Project and HapMap database (Minor Allele Frequency, MAF ≥ 1%), variants that didn’t present in these databases were considered novel^12^.

Furthermore, all patients were excluded with large and complex rearrangement mutations in *RAB39B* when analyzed by quantitative Real-Time PCR (qRT-PCR). Another 2 primer pairs were designed spanning the exon and intron of *RAB39B*, and GAPDH was selected as an internal control gene (the details of primer sequences are listed in Table 2). The concentrations of all DNA samples were diluted to be at 20–30 ng/ul, and the amplification efficiencies of the target gene and reference gene were approximately equal. qRT-PCR was performed on the Bio-Rad CFX96 in 96-well (Bio-Rad Laboratories, Lnc, United Kingdom) Multiplate^®^ PCR Plates^TM^. PCR reaction conditions were based on the standard procedures recommended by the manufacturer (Thermo Scientific, Maxima SYBR Green qPCR Master Mix (2X), #K0251). All samples and negative controls were amplified in triplicate and 2^−△△CT^ method was used to analyze the relative changes in gene expression. All the methods were carried out in accordance with the approved guidelines^8^,^13^.

## Results

We did not identify any variants in the *RAB39B* gene (including single nucleotide polymorphism, synonymous and intronic variants) among the 195 familial PD patients by Sanger sequencing. WES results also showed no variants in the whole *RAB39B* gene. Moreover, relative quantification by qRT-PCR showed that the relative copy number of *RAB39B* expression in our 195 PD individuals was between 0.8–1.2 in female PD patients and 0.45–0.6 in male PD patients, which meant that there were no dosage mutations (such as large and complex rearrangements mutations) in *RAB39B* gene among our familial PD patients.

## Discussion

The *RAB39B* gene is located at chromosome Xq28 and encodes a member of the Rab family protein, which plays a role in vesicular trafficking of eukaryotic cells. Since it was first identified in 2002, previous studies have reported that the mutations of *RAB39B* can lead to many neurological disorders, such as X-linked mental retardation (XLMR), Charcot-Marie-Tooth disease (CMT), and Warburg Micro syndrome^14^,^15^. Recently, Wilson and colleagues reported a missense mutation c.503C > A (p.Thr168Lys) and a complete deletion of *RAB39B* which was associated with a complex disease including atypical PD symptoms and intellectual disability. In addition, another mutation including c.574G > A missense mutation and c.557G > A truncating mutation were reported in different ethnic PD patients, suggesting that *RAB39B* may be a causative factor in familial PD patients. In our former study, we didn’t find any association between *RAB39B* and early-onset PD in Chinese patients^16^. Therefore, this study was conducted to further validate the role of *RAB39B* in familial PD patients among Chinese Han population (patients in these two studies have no overlapping). However, we did not find any relationship between them. In summary, our study suggested that *RAB39B* mutation is rare in Chinese PD patients, both in sporadic early onset PD patients and familial PD patients.

## Additional Information

**How to cite this article**: Kang, J.- *et al.* *RAB39B* gene mutations are not linked to familial Parkinson’s disease in China. *Sci. Rep.* **6**, 34502; doi: 10.1038/srep34502 (2016).

## Figures and Tables

**Table 1:** Primer sequences for Sanger sequencing.

Gene	Primer	Sequence
*RAB39B*	E1-F	CTGGGCCTACCCAGCTTC
	E1-R	TTCGGCAGAATCCTCAAGAC
	E2-F	GGACTGTGTCACTAACCAGGAA
	E2-R	CTGAGAGGGAGGCTCACTTG

**Table 2:** Primer sequences for quantitative Real-Time PCR.

Gene	Primer	Sequence
*RAB39B*	E1-F	AGTTCCGGCTCATTGTCATC
	E1-R	GTTTTCCTGGCTCGATCTCC
	E2-F	TTTGTTCTGGTGGGTCACAA
	E2-R	TGTGAAGGCTTTCTCCACATT
*GAPDH*	F	CAAGGTCATCCATGACAACTTTG
	R	GTCCACCACCCTGTTGCTGTAG
